# Super-Resolution-Assisted Farmland Boundary Extraction from Medium-Resolution Satellite Image: A Real-ESRGAN and YOLO Segmentation Framework

**DOI:** 10.3390/s26175613

**Published:** 2026-09-03

**Authors:** Junyao Yu, Hui Yin, Xiaofan Huang, Jiaying Liu, Shangguo Yang, Baisheng Zeng, Jiayu Zhang, Xuanyan Wang, Bo Xiong

**Affiliations:** 1 School of Geography and Tourism, Huizhou University, Huizhou 516007, China; 2412060134@stu.hzu.edu.cn (J.Y.); 2406020208@stu.hzu.edu.cn (X.H.); 2412060122@stu.hzu.edu.cn (J.L.); 2412060133@stu.hzu.edu.cn (S.Y.); 2412060135@stu.hzu.edu.cn (B.Z.); 2409010339@stu.hzu.edu.cn (J.Z.); bxiong@hzu.edu.cn (B.X.); 2 College of Mechanical and Electrical Engineering, Tarim University, Alaer 843300, China; 8011223126@stumail.taru.edu.cn; 3 College of Forestry, Wildlife and Environment, Auburn University, Auburn, AL 36849, USA

**Keywords:** farmland boundary extraction, Real-ESRGAN, YOLO segmentation framework, super-resolution, medium-resolution

## Abstract

**Highlights:**

**What are the main findings?**

- An extraction framework for fragmented farmland boundaries from medium-resolution imagery with strong generalization capability.
- A quantitative link between super-resolution reconstruction quality and downstream segmentation accuracy.

**What are the implications of the main findings?**

- A freely available alternative to high-resolution satellite data for precision agriculture monitoring in data-scarce regions.
- Bridges the gap between low-level image enhancement and high-level semantic analysis in remote sensing workflows.

**Abstract:**

This study addresses the issue of insufficient spatial resolution in remote sensing images for farmland boundary identification in precision agriculture. It proposes a framework that combines Real-ESRGAN, a GAN-based blind super-resolution algorithm, with YOLO, a real-time instance segmentation framework, to improve farmland boundary extraction accuracy from medium-resolution satellite imagery. Using GF-2 imagery of the agricultural area of Nanxiong City, Guangdong Province, a manually annotated farmland boundary dataset was constructed. The experiments were conducted in this single study area (Nanxiong City); the generalization of the proposed framework to other regions, crops, and sensor platforms requires further validation. The super-resolution preprocessing restored a 1 m resolution from 4 m input while enhancing boundary-related high-frequency details and mitigating aliasing-induced field merging. In farmland boundary recognition, the super-resolved 1 m images achieved mAP@0.5 of 0.755 and mAP@0.5:0.95 of 0.628, approaching the resampled 1 m reference (0.823 and 0.733) and clearly outperforming the resampled 4 m baseline (zero accuracy). The reported mAP values are validation-set best-checkpoint figures and therefore represent an optimistic upper bound under the current spatially autocorrelated split. The framework provides a cost-effective solution for large-scale farmland boundary extraction and precision agricultural management.

## 1. Introduction

Precise delineation of farmland boundaries is critical for crop monitoring, yield estimation, land resource management, and precision agriculture [1,2,3,4,5]. Especially in regions dominated by smallholder farming systems, farmland plots typically exhibit fragmented, irregular, and highly heterogeneous characteristics, making the boundary delineation task extremely challenging [6,7,8]. Although remote sensing images provide efficient and scalable solutions for large-area observations [9,10], medium-resolution data (e.g., the 4 m multispectral data from the GF-2 satellite) often face issues such as blurred boundaries, mixed pixels, and incomplete representation of fine-scale structures [7,11,12,13]. These limitations significantly hinder the accurate extraction of small and fragmented farmland plots [1,7,8,14]. Therefore, improving boundary delineation performance under medium-resolution remote sensing conditions remains a critical and practical issue in the field of agricultural remote sensing.

In recent years, the rapid development of deep learning has significantly improved the accuracy of farmland boundary extraction [15,16]. For example, some studies have proposed multi-scale feature learning frameworks to better capture the complex spatial features of farmlands [17,18,19]. Other researchers have further utilized attention mechanisms to enhance the representation capabilities for heterogeneous agricultural landscapes [20]. To improve boundary continuity and geometric consistency, researchers have also proposed boundary-aware and topological constraint methods [1,8,21], such as incorporating structural prior information into segmentation networks [21,22]. Instance segmentation methods based on deep learning, particularly models based on the YOLO framework, have demonstrated powerful multi-scale feature representation capabilities, enabling the precise outlining of boundary contours and the generation of high-quality instance segmentation masks [23]. However, the effectiveness of these segmentation models is still fundamentally limited by the spatial resolution of the input images [18,24,25].

At the same time, Super-Resolution (SR) technology has been widely studied for enhancing image details and improving visual quality [9,26,27,28]. Unlike traditional natural images, the degradation process of remote sensing images is usually more complex and depends on sensor characteristics [29,30]. Generative Adversarial Networks (GANs) have shown strong capabilities in reconstructing high-frequency details [31,32] and have been widely applied in the super-resolution reconstruction of remote sensing images [11,24,28]. More advanced methods have shifted toward degradation-aware modeling, aiming to better approximate real-world image degradation processes such as blurring, noise, and compression artifacts [33,34,35]. Among these, Real-ESRGAN, developed by Xintao Wang et al., introduced a high-order degradation framework to simulate complex and unknown degradation, thereby improving robustness and visual fidelity in real-world scenarios [33,34,35,36].

However, most existing studies primarily use reconstruction-oriented metrics such as Peak Signal-to-Noise Ratio (PSNR) and Structural Similarity Index (SSIM) to evaluate SR performance [26,27,28], while their effectiveness in downstream tasks—specifically, farmland boundary segmentation—remains insufficiently explored [13,25]. This reveals a critical gap between low-level image enhancement and high-level semantic understanding [18,25]. More importantly, existing research rarely establishes a direct quantitative link between SR enhancement and downstream segmentation performance, leaving the potential practical benefits in real-world agricultural applications unclear. Existing studies have approached task-oriented super-resolution through three principal strategies. The first is joint architecture optimization, in which super-resolution and segmentation networks are tightly coupled or unified into a single trainable model—for example, Salgueiro et al. proposed SEG-ESRGAN, a multi-task network that performs super-resolution and semantic segmentation simultaneously [24], and Wang et al. designed a unified SR + segmentation architecture based on similarity feature fusion [18]. While effective, this strategy requires retraining or fine-tuning the downstream detector whenever the SR model changes, which limits its plug-and-play usability with off-the-shelf detectors. The second is domain-adaptation-oriented SR, exemplified by Hong et al. [25], who introduced a task-oriented super-resolution framework that explicitly bridges the source and target domains. This line of work presupposes the existence of labeled source-domain imagery for adaptation, which is unavailable for satellite platforms like GF-2 where cross-region paired data are scarce. The third is SR-guided sample optimization, as in Jia et al. [13], who used super-resolution to enhance training samples for downstream cropland mapping. This strategy improves the downstream training set but leaves the SR model itself agnostic to the downstream task. A common limitation of all three strategies is that none explicitly couples the gradient-level structural fidelity of the super-resolved image to the downstream detector’s recognition objective, while remaining compatible with a fixed, off-the-shelf detector and a blind, real-world SR backbone. The proposed framework addresses this gap by introducing a task-coupled gradient-matching term into the Real-ESRGAN loss function, leaving both the SR backbone and the downstream detector otherwise unchanged.

To address this gap, the study treats super-resolution as a task-oriented enhancement strategy rather than an end goal [25]. The objective of the research is to improve the accuracy of farmland boundary segmentation, particularly to resolve issues of plot adhesion and boundary fragmentation caused by Modulation Transfer Function (MTF) decay and aliasing effects introduced by resampling in medium-resolution images such as GF-2 [13,37]. By adopting a super-resolution method based on the Real-ESRGAN model, the study aims to recover boundary-related high-frequency phase information (such as Laplacian zero-crossings) and spatial structures [13,33,34]. The enhanced images are subsequently used as input for downstream segmentation models to improve extraction accuracy for small and fragmented farmland plots [18,25]. By explicitly linking super-resolution performance with segmentation accuracy, the study establishes a task-driven evaluation framework that highlights the practical value of super-resolution in agricultural remote sensing. The proposed approach not only improves boundary delineation accuracy but also reduces reliance on high-resolution images [10,24], thereby providing a cost-effective and scalable solution for precision agriculture and land management [1,6,12]. We hypothesize that super-resolution preprocessing, by recovering boundary-related high-frequency information, can effectively improve farmland boundary segmentation accuracy on medium-resolution imagery.

## 2. Materials and Methods

### 2.1. Study Area Description

**Figure 1:**
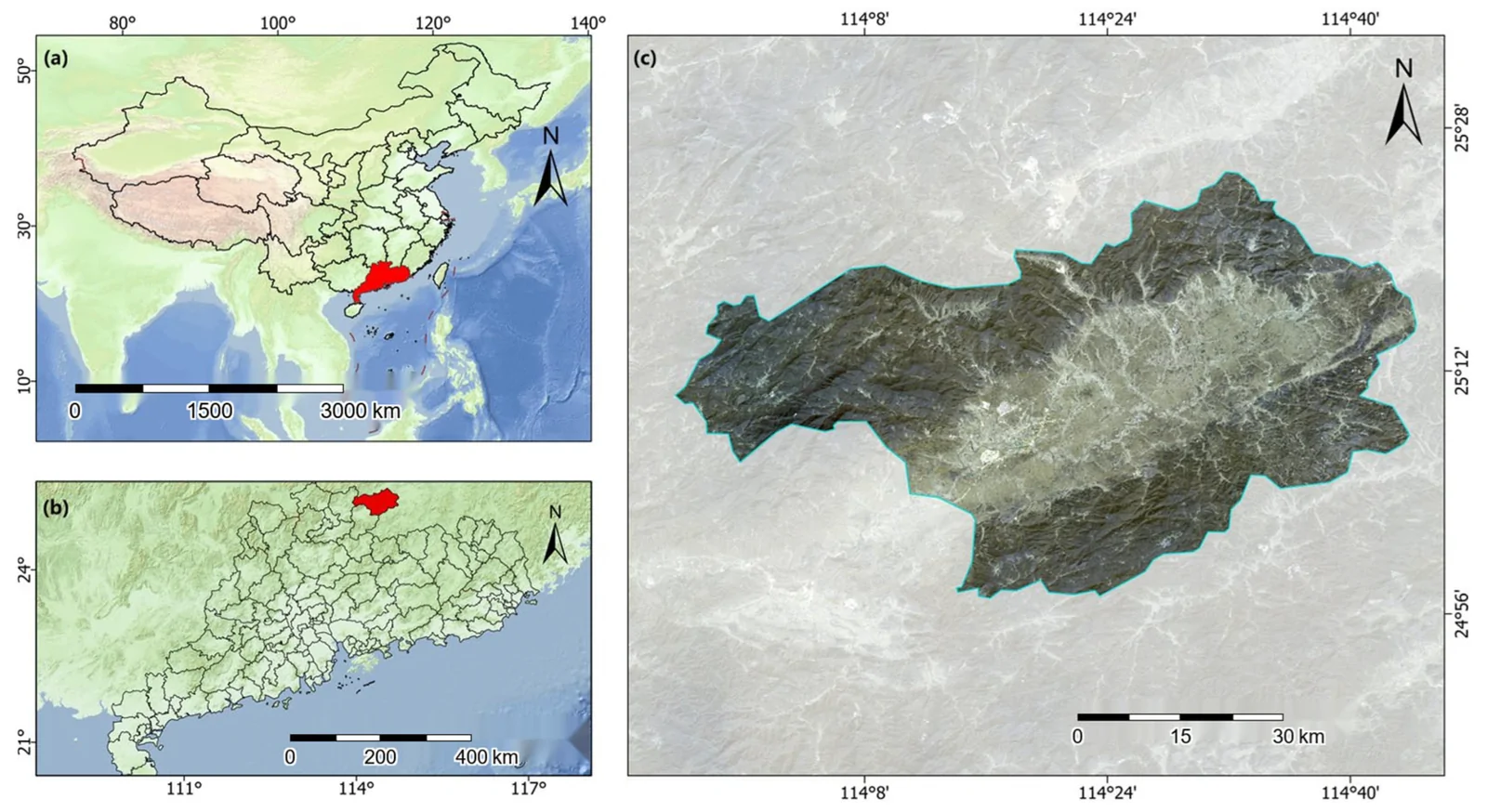
Geographic location of the study area in Nanxiong City, Guangdong Province, China. (**a**) Location of Guangdong Province in China; (**b**) location of Nanxiong City within Guangdong Province; (**c**) GF-2 imagery of the study area.

This study area is located in Nanxiong City, Guangdong Province, southern China (Figure 1). Nanxiong City is located in the mountainous region of northern Guangdong within the Zhenjiang River basin—a tributary of the upper reaches of the Beijiang River. It features a typical hilly terrain, and its agricultural development is significantly influenced by a combination of topography, climate, and water resources. As a key agricultural region on the outskirts of the Pearl River Delta, Nanxiong plays a crucial role in ensuring regional food security, with rice cultivation serving as its core industry. The region has a subtropical monsoon climate characterized by concurrent rainfall and heat, with ample annual precipitation that provides the basic growing conditions for water-intensive crops such as rice. However, due to its mountainous and hilly terrain, Nanxiong’s arable land is fragmented, the proportion of effectively irrigated land is relatively low, and the development of agricultural infrastructure faces significant challenges.

### 2.2. Data Source

The raw remote sensing image data used in this study were obtained from the official website of the China Resource Satellite Application Center, which can be found at http://gdgf.gd.gov.cn/GDGF_Portal/index.jsp (accessed on 22 March 2025). The spatial coverage of the remote sensing images includes Nanxiong City. The remote sensing images consist of four bands: B1 (Band: Blue), B2 (Band: Green), B3 (Band: Red), and B4 (Band: NIR). Other parameters of the remote sensing images are shown in Table 1.

### 2.3. Methods

This study follows the CRISP-DM (Cross-Industry Standard Process for Data Mining) methodology to organize the workflow into six stages: (1) business understanding, (2) data understanding, (3) data preparation, (4) modeling, (5) evaluation, and (6) deployment. Accordingly, business understanding and the research gap are defined in Section 1; data understanding covers the study area and GF-2 data characteristics (Section 2.1 and Section 2.2); data preparation comprises downsampling, annotation, and dataset construction (Section 2.3.2); modeling integrates the Real-ESRGAN super-resolution network and the YOLOv11x-seg segmentation network (Section 2.3.3 and Section 2.3.4); evaluation uses PSNR/SSIM and mAP metrics (Section 2.3.5 and Section 3); and deployment discusses the practical value for precision agriculture (Section 4 and Section 5). The proposed framework combines task-oriented super-resolution preprocessing with downstream boundary segmentation: the super-resolution stage is designed and trained (via a boundary-aware gradient loss) so that its output is judged by downstream segmentation accuracy rather than by PSNR/SSIM alone. The detector (YOLOv11x-seg) is used off-the-shelf, with only training-hyperparameter adjustments (learning rate, batch size, epochs, and early-stopping protocol).

#### 2.3.1. Technical Workflow of the Study

**Figure 2:**
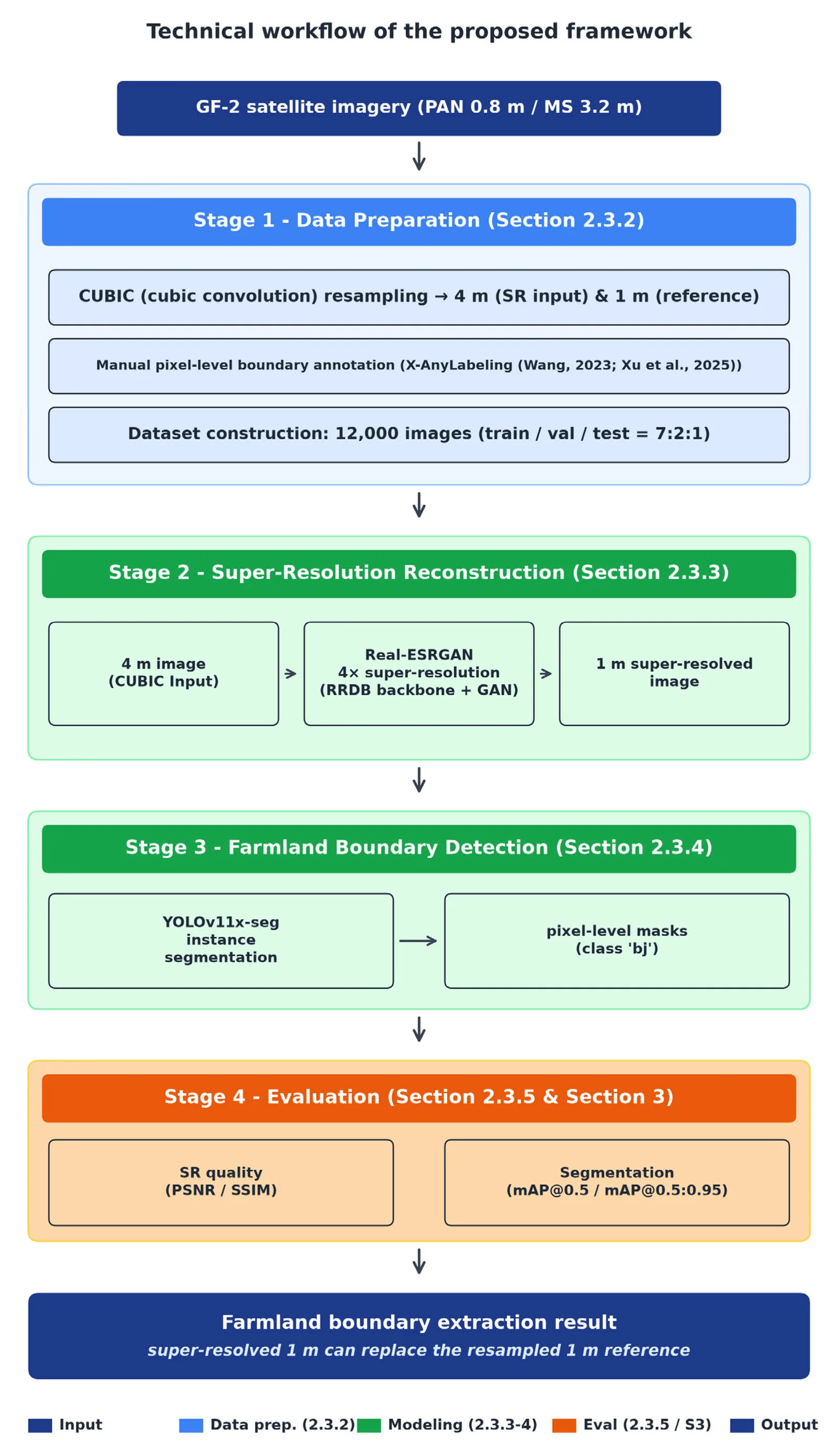
Technical workflow map of this study. Manual boundary annotation was performed with X-AnyLabeling [40,41]. SR = super-resolution; GSD = ground sampling distance; PAN = panchromatic; MS = multispectral; PSNR = peak signal-to-noise ratio; SSIM = structural similarity index; mAP = mean average precision.

**Figure 3:**
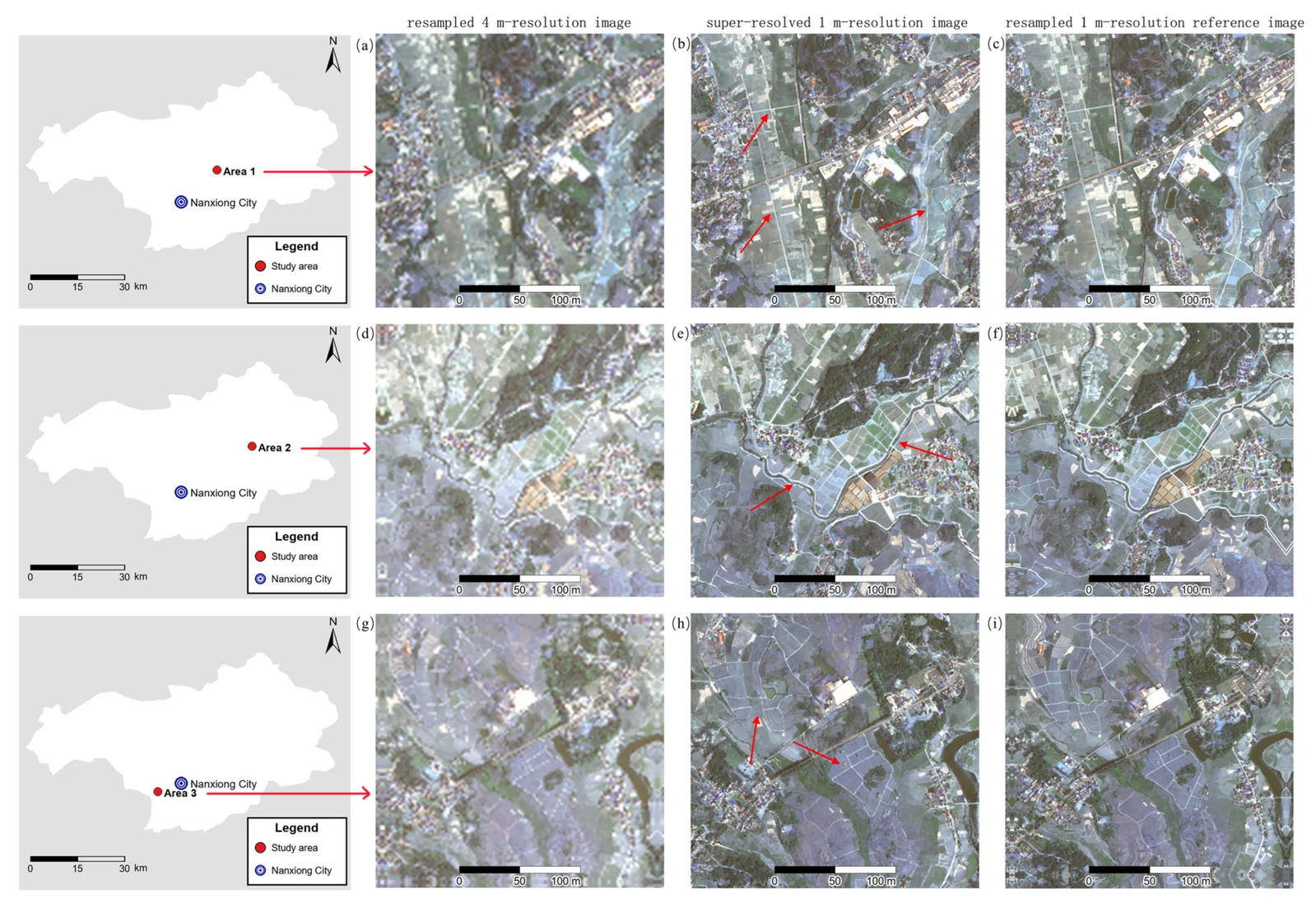
Visual comparison of super-resolution results for three representative subregions. The leftmost column depicts the location of each subregion within the study area. The images were acquired by the GF-2 satellite on 22 March 2025 (RGB composite: B3/B2/B1). The scale bar in each row indicates 100 m and applies to all three panels of that row. Each row shows the same subregion (clips 2, 70, and 52, top to bottom) at three resolutions: panels (**a**,**d**,**g**) are the 4 m input images (122 × 116 pixels); panels (**b**,**e**,**h**) are the super-resolved 1 m images (484 × 460 pixels); and panels (**c**,**f**,**i**) are the 1 m reference images (484 × 460 pixels). The 4 m panels therefore contain one quarter of the linear pixel count of the 1 m panels, consistent with the 4:1 resolution ratio. Panels (**a**,**d**,**g**) are displayed on their native 4 m grid using the default rendering kernel of the figure-compositing software, with no additional display-side smoothing.

The technical workflow of this study is shown in Figure 2. Firstly, the 0.8 m resolution raw GF-2 data were downsampled to 1 m and 4 m resolutions using CUBIC (cubic convolution) interpolation in ArcMap (Version 10.8), respectively. Then, the Real-ESRGAN super-resolution module was used to upscale the downsampled 4 m resolution image to 1 m resolution. Next, the YOLOv11x-seg image segmentation module was used to compare the super-resolved 1 m resolution image with the resampled 1 m resolution image. Finally, validation results demonstrate that the super-resolved 1 m resolution image can replace the resampled 1 m resolution reference image for identifying farmland boundaries. Bicubic interpolation applies an implicit anti-aliasing low-pass filter (a 4 × 4 cubic-kernel convolution); the procedure is therefore a standard, reproducible, non-adversarial simulation of a coarser sensor’s instantaneous field of view, rather than an unfiltered decimation. For independent verification, the pixel dimensions of each panel (the 4 m panels contain one quarter of the linear pixel count of the 1 m panels, consistent with the 4:1 resolution ratio) and the display kernel (no display-side smoothing beyond default rendering) are shown in Figure 2. The authors have submitted the 4 m GeoTIFF together with the downsampling script as Supplementary Materials. Specifically, the downsampling was implemented with the Resample tool of ArcGIS Desktop (version 10.8) (ArcToolbox—Data Management Tools—Raster Processing—Resample) [38,39]: the 0.8 m panchromatic band served as the input raster; the output cell size was set to 1 m and 4 m in two independent runs; and the resampling technique was set to CUBIC (cubic convolution), which estimates each output pixel as a weighted combination of the sixteen nearest input pixels through a 4 × 4 cubic convolution kernel. The output GeoTIFF rasters share the same coordinate reference system and geographic extent as the source image, so that the 1 m and 4 m products are pixel-registered with each other and with the 0.8 m source. For the qualitative assessment in Figure 2, three representative subregions (clips 2, 70, and 52) were cropped from the two downsampled products using the Clip tool of ArcGIS Desktop (Version 10.8), producing the 484 × 460-pixel (1 m) and 122 × 116-pixel (4 m) panels shown in Figure 3.

#### 2.3.2. Data Preprocessing and Annotation

In remote sensing images, farmland boundaries often exhibit irregular shapes, narrow widths, and high levels of overlap with surrounding land features, making pixel-level boundary annotation particularly challenging. When boundaries are blurred or there are transitional areas between adjacent parcels, differences in judgment can easily arise among annotators, thereby affecting annotation consistency. Furthermore, the identification of farmland boundaries relies heavily on their contextual environment; without information from surrounding land features, semantic ambiguity can easily arise. Prolonged repetitive annotation may also lead to annotator fatigue, further reducing annotation accuracy [42,43,44]. Under large-scale remote sensing image conditions, the aforementioned issues significantly increase the time cost of manual annotation and the difficulty of quality control.

Annotations were performed manually by a single expert annotator (the first author) using X-AnyLabeling version 2.1.3 [40,41], with cross-validation against field photographs. The reference images are the bicubic-resampled 1 m panchromatic images (0.8 m → 1 m via CUBIC interpolation in ArcMap (Version 10.8)), which serve as the ground-truth target of the super-resolution network. No pre-trained model was used for any auxiliary screening. The 0.8 m raw images were not retained after downsampling; their use as cross-validation would have been preferable and is noted as a limitation. In this work, ‘boundary width’ denotes the effective boundary width—the width of the visible terrace-edge transition zone that includes the field-platform margin—rather than the narrow physical ridge structure; for the terraced fields in Nanxiong, the average effective boundary width is approximately 3 m. The order of magnitude of this width is supported by the Guangdong Modern Standard Farmland Construction Standard (Trial) [45], which stipulates that, after land consolidation, the leveled terrace platform should be at least 3 m wide and the terraced-field ridge should be 0.4–0.6 m wide and no less than 0.3 m high; field surveys in Nanxiong are consistent with these images, yielding an average effective boundary width of approximately 3 m (including the platform margin), while the actual physical ridge is only 0.4–0.6 m. The dataset used for super-resolution reconstruction consisted of 12,000 images, which were strictly divided into training, validation, and test sets in a 7:2:1 ratio to ensure the scientific rigor of model training and the objectivity of evaluation.

#### 2.3.3. Super-Resolution Reconstruction Module

Real-ESRGAN (Real-Enhanced Super-Resolution Generative Adversarial Network) is a super-resolution model developed from ESRGAN [46,47], designed to address complex and unknown image degradation issues in real-world scenarios [48,49]. Unlike traditional super-resolution models that rely on paired low- and high-resolution samples, Real-ESRGAN constructs a high-order degradation model to simulate various real-world degradation processes (such as blurring, downsampling, and combinations of noise and compression artifacts) during the training phase, thereby enhancing the model’s adaptability to the degradation patterns found in actual remote sensing images [50,51,52,53,54].

The model adopts a generative adversarial network framework, consisting of a generator and a discriminator [55]. The generator takes low-resolution images as input and outputs corresponding high-resolution reconstructions; its core structure is the Residual-in-Residual Dense Block (RRDB) [56]. Through dense connections and multi-level residual learning, the RRDB effectively mitigates the vanishing gradient problem while increasing network depth, enabling the model to more fully capture local textures and fine-scale features in images [57]. The capability is particularly crucial for fine-scale structures commonly found in remote sensing images, such as field ridges and ditches [58,59,60,61,62,63].

In terms of discriminator design, Real-ESRGAN adopts a U-Net-based architecture to evaluate the generated results at the pixel level. Compared to traditional discriminators, the architecture provides more refined gradient feedback for local regions, which helps improve the reconstruction quality of edge regions [64,65,66]. To improve training stability, the model further incorporates strategies such as spectral normalization to suppress instability during training. The loss function is composed of pixel-level loss, perceptual loss, and adversarial loss, ensuring both structural consistency and visual realism.

In the implementation process, this study adjusted the model training strategy to accommodate limited computational resources. During training, 512 × 512-pixel high-resolution image patches are used, and a small learning rate is set to ensure the stability of parameter updates. Additionally, mixed-precision training and gradient clipping strategies are introduced to reduce GPU memory consumption and prevent gradient vanishing. To enhance the model’s responsiveness to boundary information, a gradient loss term is incorporated into the loss function, and its weight is appropriately increased to strengthen the constraints on changes in the image’s edge regions. Formally, the boundary-aware gradient loss is defined as the L1 penalty between the spatial gradient fields of the super-resolved image I_SR and of its high-resolution reference I_HR, with weight λ_grad = 0.3; the full expression is given in Equation (1) below. The remaining Real-ESRGAN loss terms (pixel L1, perceptual VGG, and U-Net adversarial losses) and their relative weights are inherited unchanged from the official Real-ESRGAN recipe, so the total objective of the super-resolution stage becomes L_total = L_pix + L_percep + λ_adv·L_adv + λ_grad·L_grad, with λ_grad = 0.3. A controlled ablation isolating the contribution of the L_grad term was not conducted in this revision because re-training the SR model under matched compute budgets would exceed the present compute budget; we therefore support the L_grad contribution with the validation-set PSNR/SSIM behavior in Section 3.1.1 only, and document the absence of an ablation as a limitation in Section 4.3.

(1) $$Lgrad=\lambda \;\cdot \;E\;x\;\left\|\nabla I_{SR}\left(x\right)-\nabla I_{HR}\left(x\right)\right\|1,\;\lambda =0.3$$

where Lgrad denotes the gradient loss term, λ is the weighting coefficient (set to 0.3), E_x represents the expectation over all spatial locations x, ∇I_SR(x) and ∇I_HR(x) are the spatial gradients of the super-resolved image and the high-resolution reference image, respectively, and ‖1 is the L1 norm.

It should be emphasized that in this study, super-resolution reconstruction is not treated as an independent image enhancement task but rather as a preprocessing step for farmland boundary recognition. By enhancing the spatial detail representation of the images—particularly the clarity and continuity of boundary regions—it provides more discriminative input features for subsequent boundary recognition models.

#### 2.3.4. Farmland Boundary Detection Module

Convolutional neural networks (CNNs) learn hierarchical visual representations through stacked convolutional layers, non-linear activations (e.g., SiLU/Swish), and progressive spatial downsampling. Each convolutional layer applies a set of learnable kernels to extract local patterns over a prescribed receptive field; the inter-layer non-linearity enables the network to compose simple low-level features (edges, gradients) into increasingly abstract mid- and high-level patterns (textures, parts, objects). This hierarchical feature-extraction paradigm is well suited to remote-sensing imagery, where the spatial scale of targets spans from a few pixels (field ridges) to hundreds of pixels (parcel blocks): shallow layers capture micro-textures and high-frequency edge cues critical for boundary localization, while deep layers encode parcel-level semantics and shape priors. To balance representational capacity with computational cost, modern CNN backbones incorporate residual connections, depth-wise separable convolutions, and channel-attention modules. After completing super-resolution reconstruction, this study employs the segmentation model of YOLOv11x-seg to automatically detect farmland boundaries [67]. The YOLO family has evolved through successive architectural refinements, from YOLOv3’s Darknet-53, through YOLOv5’s C3 module and YOLOv8’s C2f block, to the recent YOLOv11 backbone, which adopts a redesigned C3k2 (cross-stage partial with kernel size 2) block for finer-grained feature aggregation, a C2PSA (cross-stage partial with position-sensitive attention) module to inject spatial-attention cues at the detection neck, and a simplified SPPF (spatial pyramid pooling–fast) head. These refinements collectively improve small-object localization and elongated-structure detection, two properties directly relevant to fragmented farmland boundaries. In this study, the YOLOv11x-seg model is adopted off-the-shelf: the backbone, neck, and segmentation head are used from the official Ultralytics release [68,69], and the segmentation branch is the only branch retained at inference, with no architectural modification.

In this study, farmland boundaries are treated as continuous spatial structures, and their identification relies on the comprehensive modeling of local texture variations and elongated boundary features. Because YOLOv11x-seg incorporates an off-the-shelf segmentation branch, it directly outputs pixel-level boundary masks, thereby avoiding the localization errors associated with relying solely on bounding boxes. Furthermore, while maintaining high inference efficiency, the model can adapt to resolution variations in super-resolution images, making it suitable for the batch processing of large-scale remote sensing images.

Regarding the model training details, the model was trained for a maximum of 500 epochs with a batch size of 8 and an input resolution of 512 × 512, but an early stopping strategy was applied to halt training when validation performance ceased to improve (approximately around epoch 100). Stochastic Gradient Descent (SGD) was used as the optimizer, with an initial learning rate of 0.01 and a momentum of 0.9. Weight decay was selectively applied to different parameter groups according to the default Ultralytics configuration. Additionally, mixed training was conducted by combining local farmland samples with multi-regional farmland images to enhance the model’s adaptability to morphological differences in farmland boundaries across various geographic environments. Training was performed on a single GPU, with GPU memory usage of about 6 GB. Through these settings, the boundary recognition model can learn representative boundary features in both regular and irregular farmland scenarios, providing stable outputs for subsequent performance evaluation.

#### 2.3.5. Evaluation of Super-Resolution Models

To systematically assess the impact of super-resolution preprocessing on the farmland boundary detection task, this study established a comprehensive experimental workflow comprising “super-resolution reconstruction—boundary detection—result evaluation.” Firstly, a 4 m resolution GF-2 image obtained by CUBIC (cubic convolution) interpolation in ArcMap (Version 10.8) was used as input and subjected to super-resolution reconstruction to obtain a 1 m resolution image. Subsequently, the same boundary detection model was applied to both the super-resolved image and the resampled 1 m reference image, creating a control group and an experimental group.

Regarding the design of evaluation metrics, this study addresses two aspects: image reconstruction quality and the performance of downstream tasks. For the super-resolution reconstruction results, common metrics such as PSNR and SSIM were used to evaluate reconstruction quality at the pixel and structural levels [70]. Additionally, in line with the requirements of the boundary detection task, a qualitative analysis was conducted on image edge sharpness and visual continuity.

For farmland boundary recognition results, manually annotated data served as the reference standard. Metrics such as Precision and mAP were primarily used to evaluate the model’s performance in boundary localization and overall recognition accuracy. In particular, the mean Average Precision at IoU threshold 0.5 (mAP@0.5) and the mean Average Precision averaged over IoU thresholds from 0.5 to 0.95 (mAP@0.5:0.95) use the IoU threshold as the benchmark for object detection models in bounding boxes [71].

To objectively evaluate the performance of the super-resolution model, this study adopted PSNR and SSIM as key evaluation metrics. PSNR measures the pixel-level error between the reconstructed image and the reference high-resolution image. The formula for PSNR is defined as follows.

(2) $$\mathrm{PSNR}=10\log_{10}\frac{L^{2}}{\mathrm{MSE}}$$

where *L* represents the maximum pixel value, and *MSE* stands for mean squared error; a higher value indicates less reconstruction distortion.

The formula for *SSIM* is defined as follows:

(3) $$\;\mathrm{SSIM}\left(x,y\right)\;=\;\frac{\left(2\mu_{x\mu_{y}}+{\;c}_{1}\right)\left(2\sigma_{\left\{\mathrm{xy}\right\}}+c_{2}\right)}{\left(\mu_{x}^{2}+\mu_{y}^{2}+c_{1}\right)\left(\sigma_{x}^{2}+\sigma_{y}^{2}+c_{2}\right)}$$

where *μ_x_* and *μ_y_* represent the local mean pixel brightness of the x and y components of the images, respectively, $\sigma_{x}^{2}$ and $\sigma_{y}^{2}$ represent the local variances of the pixel brightness in the images x and y, respectively, and $\sigma_{\left\{\mathrm{xy}\right\}}$ represents the local covariance in the images x and y. *c*_1_ and *c*_2_ represent the constant terms, respectively. The metric evaluates the similarity between the reconstructed image and the reference image in terms of luminance, contrast, and structure using the mean μ, standard deviation σ, and covariance $\sigma_{\mathrm{xy}}$. The closer the *SSIM* is to 1, the higher the fidelity of the reconstruction result in terms of human visual perception. Both metrics are used together to evaluate the performance of the reconstruction results in terms of numerical accuracy and overall structure preservation.

For comparison, this study additionally employs EDSR (Enhanced Deep Super-Resolution) as a classical CNN-based super-resolution baseline. EDSR, introduced by Lim et al. [72], removes batch normalization from the residual network and adopts a large number of residual blocks to reconstruct high-resolution images; here it is applied through the OpenCV image-processing library (DNN_superres module) [73] as a non-GAN baseline. The same evaluation workflow and metrics (PSNR, SSIM, and downstream mAP) are applied to EDSR + OpenCV, so that the Real-ESRGAN results can be compared both numerically and visually with a representative non-generative super-resolution method (Section 3.3).

## 3. Results

### 3.1. Super-Resolution Quantitative Results and Analysis

#### 3.1.1. Quantitative Evaluation Results for PSNR and SSIM

On the reconstruction held-out subset (2400 of the 12,000 reconstructed tiles), the PSNR of the reconstructed images ranged from 24.61 to 28.45 dB, with an average of 26.19 dB; the SSIM ranged from 0.7306 to 0.8490, with an average of 0.7676. Overall, most of these reconstructed tiles maintained a relatively stable level of structural similarity, indicating that Real-ESRGAN maintains stable structural similarity across heterogeneous agricultural scenes; that is, its robustness here refers to the stability of reconstruction quality (pixel- and structure-level fidelity) rather than geographic or environmental generalization.

These 2400 tiles were used by the SR stage as its early-stopping partition and are disjoint from the 1200-tile detector test partition; the two stages’ evaluation subsets therefore share no inference samples, so the SR reconstruction metrics reported here remain out-of-sample with respect to the downstream segmentation task. A fully disjoint held-out SR test partition was not constructed in this revision because downstream segmentation accuracy, not per-pixel reconstruction fidelity, is the primary task metric of this work; this trade-off is documented here for transparency.

#### 3.1.2. Results and Analysis of Super-Resolution Qualitative Assessment

As can be seen in Figure 3, each row corresponds to the same geographic area, while each column shows different input resolutions: (a, d, g) resampled 4 m resolution image, (b, e, h) super-resolved 1 m resolution image generated by the proposed method, and (c, f, i) resampled 1 m resolution reference image. Red arrows indicate regions where the proposed super-resolution approach effectively enhances boundary clarity and preserves fine structural details, yielding visual quality comparable to a true high-resolution image.

Compared to the resampled 4 m resolution image, the 1 m resolution image obtained through super-resolution exhibits clearer contours and more continuous edge patterns in areas with linear structures, such as farmland boundaries, field ridges, and roads. Boundaries that were blurred in the image due to resolution limitations are significantly enhanced in the super-resolved results. The improvement in boundary clarity aligns with the design objective of introducing gradient loss and increasing its weight during model training. By imposing constraints on areas with significant variations in image grayscale, the model’s ability to respond to edge information during reconstruction is enhanced. From a structural perspective, the super-resolved image maintains the macro-level consistency of the scene while improving the discernibility of local details, without exhibiting large-scale structural distortions or obvious artifacts.

### 3.2. Results and Analysis of Farmland Boundary Detection

#### 3.2.1. Model Training Results and Accuracy Interpretation

**Figure 4:**
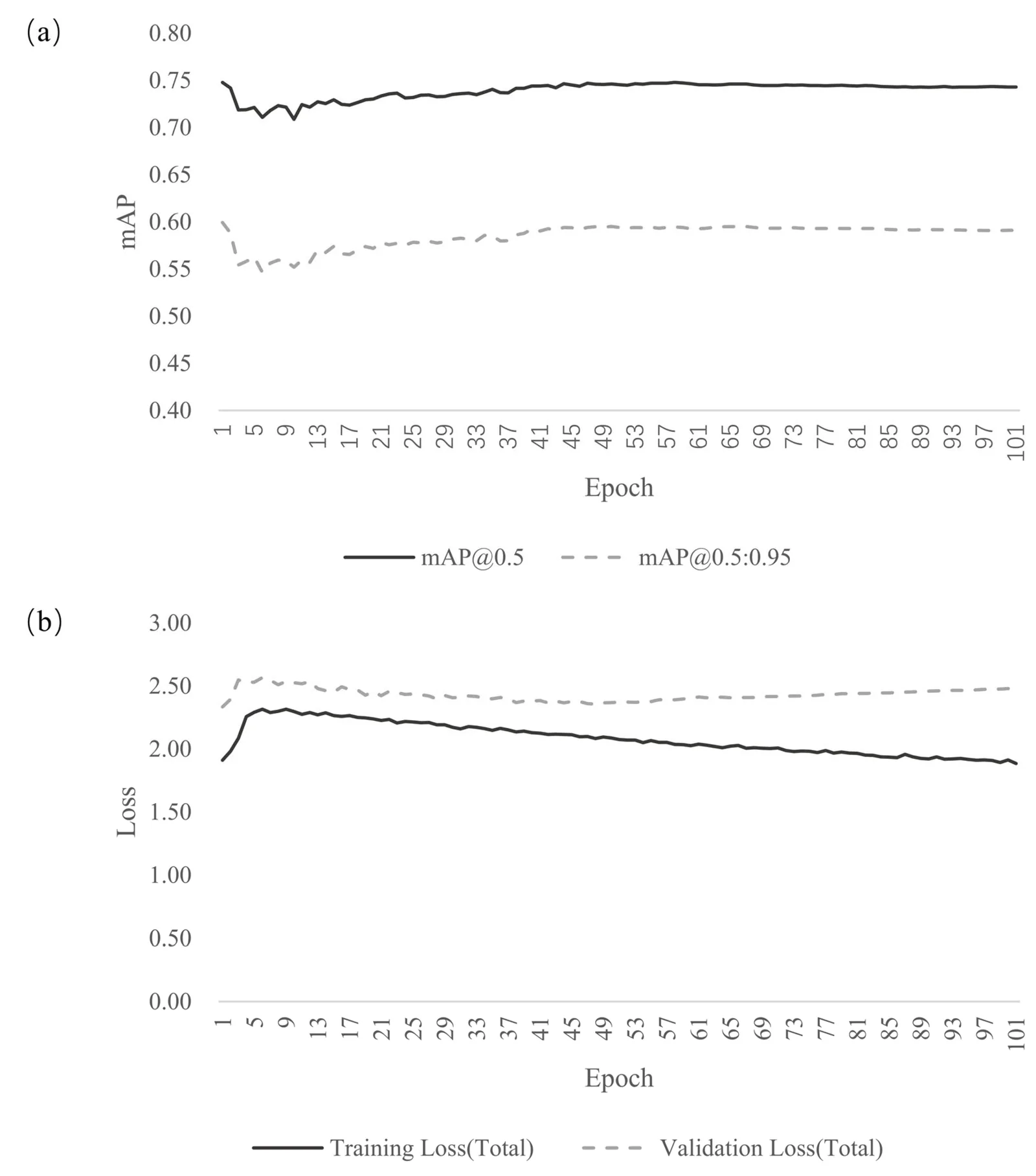
Training curves of the YOLOv11x-seg model on a super-resolved image. (**a**) Variation of mAP@0.5 and mAP@0.5:0.95 across training epochs. (**b**) Variation of total training loss and total validation loss across training epochs.

After obtaining a super-resolution image of 1 m resolution, this study conducted experiments on the automatic identification of farmland boundaries using the YOLOv11x-seg model [74]. The model training results of the YOLOv11x-seg model are shown in Figure 4. In the farmland boundary recognition task, the values of mAP@0.5 were 0.755, the values of mAP@0.5:0.95 were 0.628, and the training and validation losses were around 2.00 and 2.50, respectively. The validation results indicated that the model did not overfit and possessed good generalization ability. The detection dataset comprises 12,000 image patches generated by the Split Raster tool in ArcMap (Version 10.8) (tile-size mode, fixed-size non-overlapping tiles) over a single contiguous study area (the Nanxiong farmland mosaic); a 7:2:1 (train/val/test) partition was applied at the tile level, and the full mosaic reassembles exactly with no pixel overlap between neighbouring tiles. The test partition was strictly held out during training (train used for gradient updates, val for early stopping/model selection, test never fed to the model). Model selection and early stopping remained on the validation set; the reported mAP@0.5 = 0.755 and mAP@0.5:0.95 = 0.628 are validation-set best-checkpoint figures. An independent evaluation on the held-out test subset yields test mAP@0.5 = 0.68 and test mAP@0.5:0.95 = 0.613; both are lower than the validation-set best-checkpoint figures (0.755/0.628), so the reported values remain an optimistic upper bound under the present (spatially autocorrelated) split.

In particular, this study employs a segmentation mask-based method to model mAP@0.5:0.95 and mAP@0.5. The training and validation loss curves illustrate the model’s convergence behavior and generalization performance. Due to the Early Stopping strategy (a technique used during model training to prevent overfitting and improve model generalization), training was halted after approximately 100 epochs, and the weights saved when the model achieved optimal performance on the validation set were used for testing. The training curve shows that the model’s training loss gradually decreases with each iteration, indicating that the model fits the training data well; the validation loss exhibits an overall downward trend but with some fluctuations. These fluctuations are likely attributable to the relatively large batch size of 8 combined with the fixed learning rate (initial 0.01, SGD without a learning-rate schedule), which produces noisier gradient estimates on heterogeneous agricultural scenes; the early-stopping strategy (halting around epoch 100) prevented overfitting despite these fluctuations.

In terms of segmentation accuracy, the model of mAP@0.5:0.95 and mAP@0.5 shows minimal variation between the early and final stages of training, remaining relatively stable overall. This indicates that the model did not exhibit significant overfitting or performance degradation in the farmland boundary recognition task. For model selection and early stopping, this study used the highest boundary recognition accuracy on the validation set; the unbiased performance estimate is given by the held-out test figures reported above (test mAP@0.5 = 0.68, mAP@0.5:0.95 = 0.613), which are lower than the validation best-checkpoint values. (i.e., the mAP@0.5 = 0.755 and mAP@0.5:0.95 = 0.628 figures are validation-set best-checkpoint results; see the optimism caveat above).

#### 3.2.2. Comparative Analysis of Recognition Results

**Figure 5:**
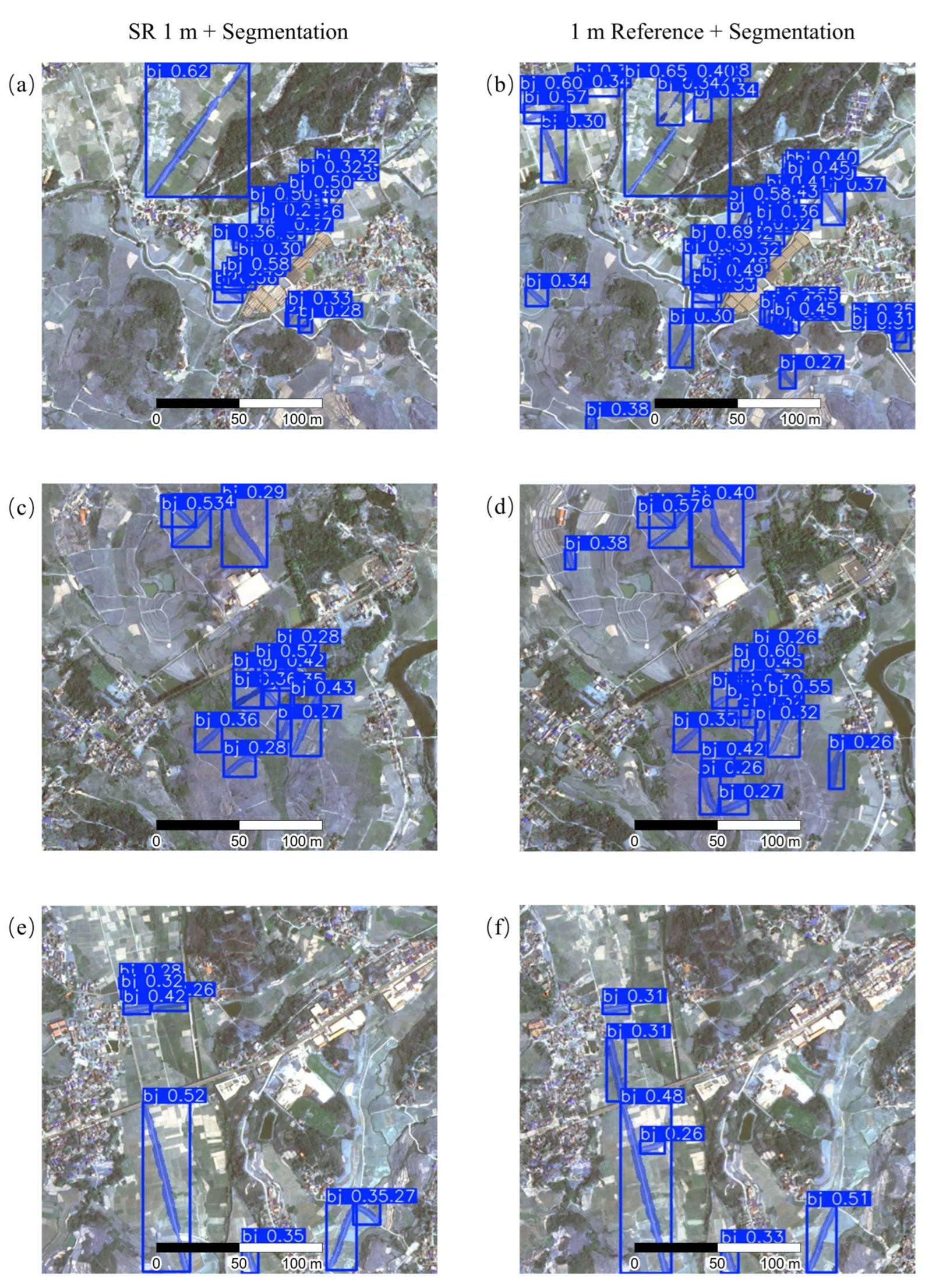
Comparison of the farmland boundary segmentation results between the super-resolved and resampled 1 m reference images. (**a**,**c**,**e**) Segmentation results obtained from the super-resolved 1 m image for three representative subregions; (**b**,**d**,**f**) segmentation results obtained from the resampled 1 m reference image for the same three subregions. In the detection visualizations, the blue bounding boxes denote the farmland boundaries identified by the model; the label “bj” indicates the boundary class, and the associated numeric value (e.g., 0.62) is the detection confidence, i.e., the model’s credibility score for that prediction.

To further evaluate the practical effectiveness of super-resolution images in identifying farmland boundaries, this study conducted a comparative analysis of the model’s segmentation results on a 1 m resolution super-resolved image versus a resampled 1 m resolution reference image of the same area (Figure 5).

Each row corresponds to the same geographic subregion, while the two columns show segmentation results obtained from a super-resolved 1 m resolution image (Figure 5a,c,e) and a resampled 1 m reference image (Figure 5b,d,f), respectively. The high consistency of boundary localization and shape between the two inputs demonstrates that the proposed super-resolution approach effectively preserves structural information critical for downstream farmland boundary segmentation.

Overall, the farmland boundaries extracted under both input conditions exhibit a high degree of consistency in terms of spatial location and overall contour, indicating that super-resolution processing has largely preserved the key information required for boundary identification. In local areas, particularly where field parcels are highly fragmented or where boundaries intersect with other land features, the recognition results on the super-resolution image still exhibit some discrepancies compared to the 1 m reference image. For example, the boundary continuity of some narrow field ridges is slightly weaker, and minor shifts occur in a few complex boundary areas. These discrepancies primarily stem from insufficient spatial information in the resampled 4 m resolution image, making it difficult for the super-resolution process to fully recover all high-frequency details.

Despite these local discrepancies, from an application perspective, the farmland boundaries derived from the super-resolution image have achieved a usable level in terms of overall structural integrity and positioning accuracy, with limited impact on tasks such as farmland delineation and area estimation. The comprehensive results indicate that, in the absence of a true high-resolution remote sensing image, the “super-resolution reconstruction + boundary recognition” workflow proposed can extract farmland boundary information of practical value from freely available medium-resolution data, providing a cost-effective technical approach for large-scale farmland monitoring and refined management.

One may ask why simpler line-detection methods, such as the linear Hough transform, are not used instead of a deep segmentation model. The Hough transform detects straight lines in edge maps and is well suited to long, rectilinear features; however, farmland boundaries in Nanxiong are typically curved, fragmented, and partially occluded by vegetation or terrain, so they cannot be approximated reliably by straight line segments. Moreover, Hough-based methods require a separate edge-detection step and output line segments rather than closed parcel polygons, which is insufficient for area estimation and cadastral purposes. The learned segmentation branch of YOLOv11x-seg, in contrast, directly produces pixel-level boundary masks that preserve curved and discontinuous boundaries, as demonstrated in Figure 5.

**Figure 6:**
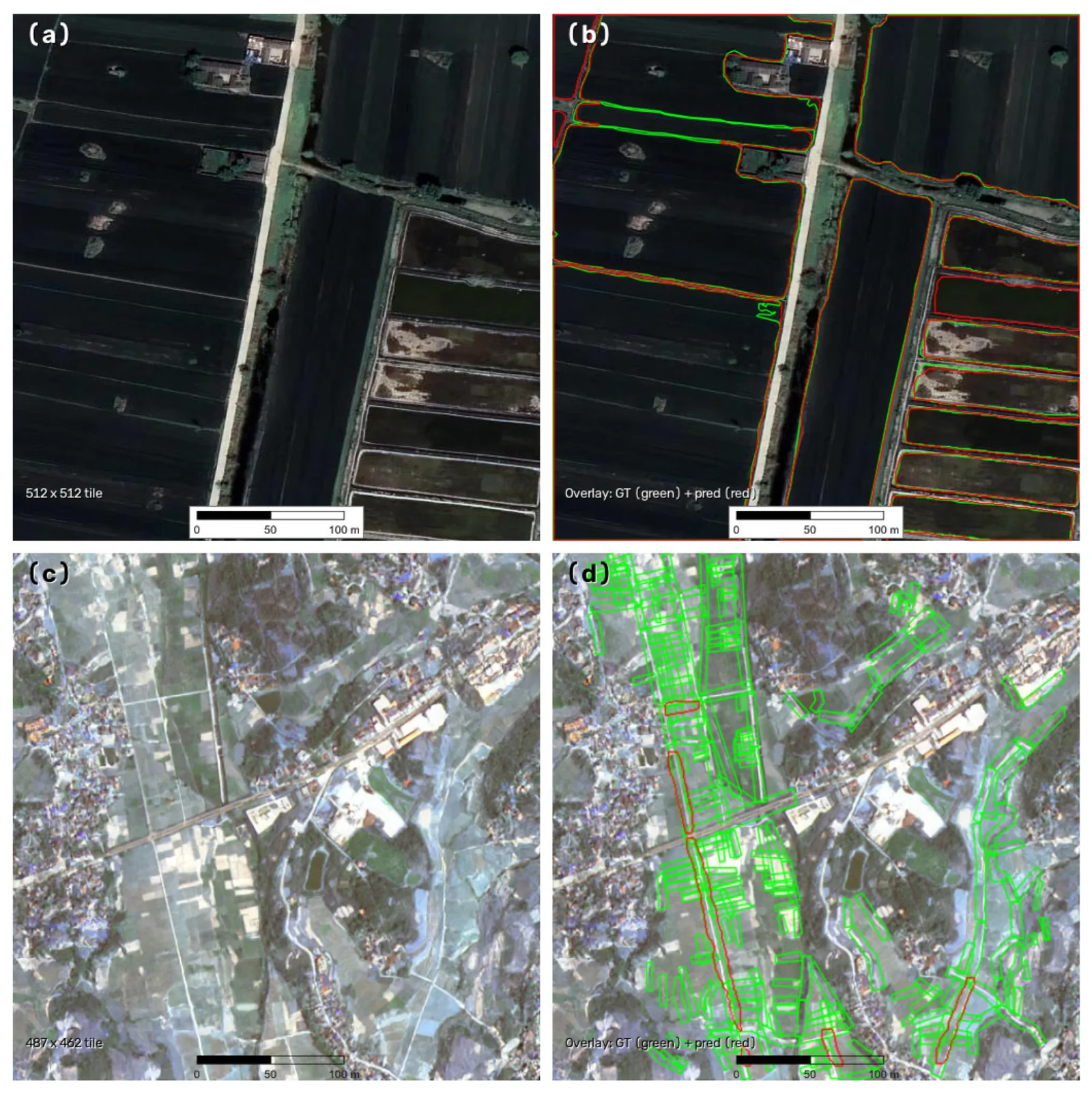
Illustration of boundary positional accuracy on SR-reconstructed 1 m tiles. (**a**) Representative 512 × 512 tile. (**b**) Overlay of manually annotated ground-truth boundaries (green) and detector-predicted boundaries (red) for the same tile; the two sets coincide to within a few metres. (**c**) A 487 × 462 tile of the type used in the EDSR comparison of Section 3.3, where detector recall is lower. (**d**) Overlay of ground-truth (green) and predicted (red) boundaries for the 487 × 462 tile, illustrating the domain-degradation effect discussed in Section 3.2.2.

To evaluate the effectiveness of the proposed super-resolution method, object detection was performed under different input conditions, with the experimental results shown in Table 2 and Figure 6. Since the detector could not identify objects in the resampled 4 m resolution image, the values of mAP@0.5 and mAP@0.5:0.95 were 0 in all cases. This outcome is attributable primarily to the physical resolution limit (the ~3 m effective boundary width is sub-pixel at 4 m GSD, below the Nyquist limit—such boundaries are not resolvable even by manual interpretation), with the 1 m → 4 m domain mismatch as a secondary factor (the 4 m image was fed to the detector by tiling at the same patch size (487 × 462) used at 1 m training, and no retraining was performed at the 4 m scale; detection used the same sliding-window protocol as at 1 m). The Nyquist sub-pixel argument below reinforces that even the best-case 4 m boundary signal is below detectable thresholds; it is not a limitation of the method per se. As stipulated by the Guangdong Modern Standard Farmland Construction Standard (Trial) [45], terraced-field ridges are 0.4–0.6 m wide and no less than 0.3 m high, while the leveled terrace platform is at least 3 m wide; consequently the effective boundary width in Nanxiong is on the order of 3 m, whereas the actual physical ridge is only 0.4–0.6 m. At a 4 m GSD a 3 m transition occupies fewer than one pixel (≈0.75 pixel) and the 0.4–0.6 m ridge occupies merely 0.10–0.15 pixels, so both fall below the Nyquist resolution limit (a feature of width W requires GSD ≤ W/2 for reliable discrimination); the boundaries therefore become physically indistinguishable from the surrounding farmland (the Nyquist limit constrains the resolvability of a ridge’s width, not the detectability of the radiometric step between adjacent parcels) [6,45]. After applying super-resolution reconstruction, detection performance improved significantly, with mAP@0.5 and mAP@0.5:0.95 reaching 0.755 and 0.628, respectively. This indicates that the proposed method can effectively recover the key visual features required for object detection. To confirm that recovered boundaries are geometrically faithful rather than merely visually plausible, a polygon-level mean boundary distance (MBD) analysis between predicted and reference outlines was therefore conducted on a held-out set of SR-reconstructed tiles for which polygon-level ground truth was available, and the results are summarized below and visualized in Figure 6. At the operating confidence of 0.25, the predicted boundaries lie on average 6.26 ± 3.54 m from the nearest manually annotated boundary (prediction-to-reference MBD) and 4.28 ± 2.56 m in the symmetric measure (range 1.89–8.24 m), with a mean intersection-over-union of 0.95 against the reference polygons. These values indicate that, where the detector fires, the recovered boundaries coincide with the reference annotations to within a few metres; the 4 m imagery simply contains no resolvable boundary signal (the ~3 m effective boundaries are sub-pixel at 4 m GSD), so recall is necessarily zero at 4 m; the super-resolution step does not induce geometrically erroneous boundary hallucination.

**Figure 7:**
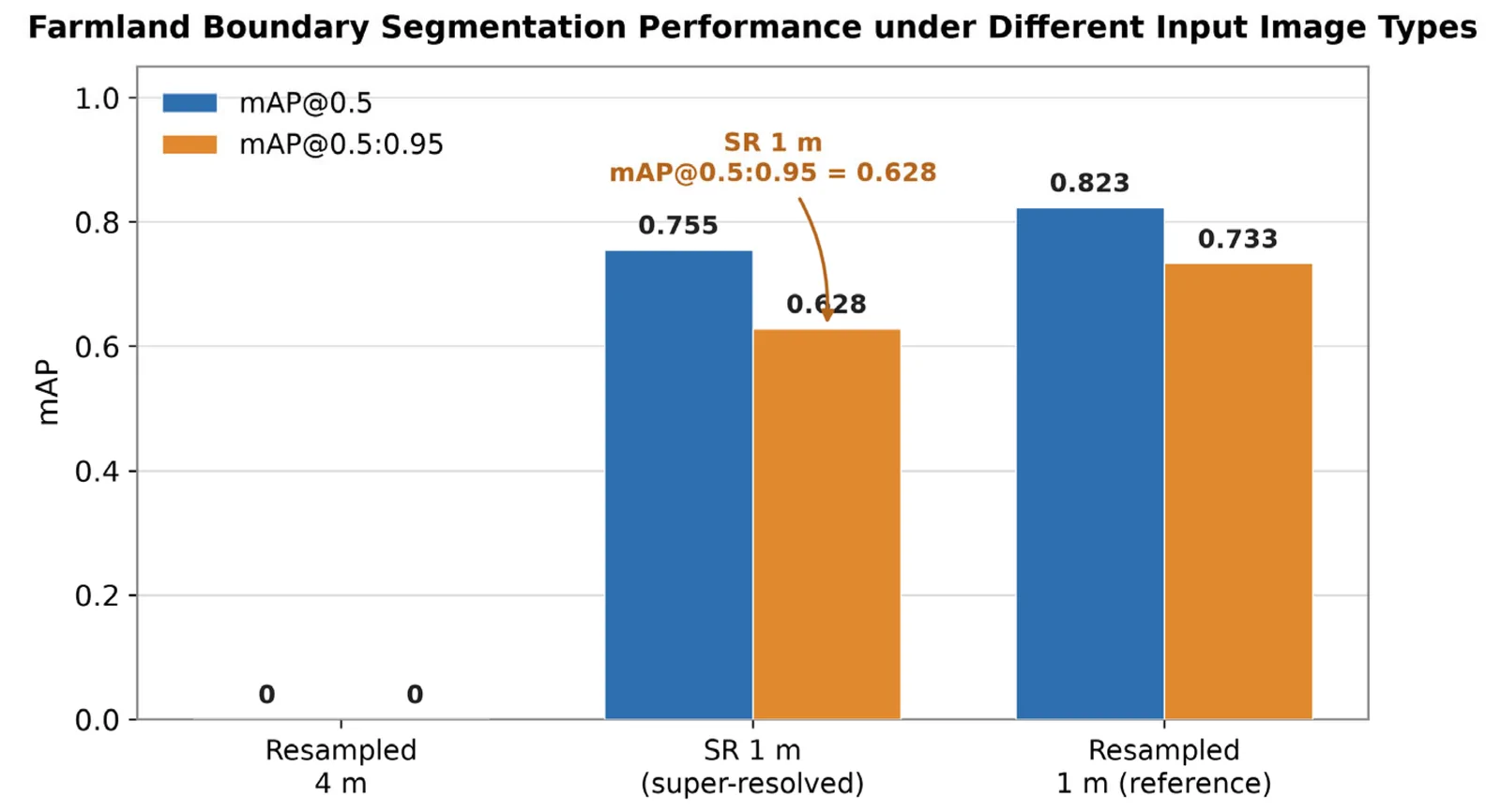
Quantitative comparison of farmland boundary segmentation accuracy under three input conditions.

As shown in Table 2 and Figure 7, the comparison covers a resampled 4 m image, a super-resolved 1 m image, and a resampled 1 m reference image of the same area. The super-resolved 1 m input attains mAP@0.5:0.95 of 0.628, whereas the 4 m input yields zero accuracy because the average boundary width of Nanxiong terraced fields (~3 m) falls below the Nyquist resolution limit at a 4 m ground sampling distance and because the 1 m trained detector was not retrained for the 4 m scale (domain mismatch), demonstrating the necessity of super-resolution for sub-pixel boundary extraction. Compared with the resampled 4 m resolution image, the 1 m resolution image reconstructed using super-resolution techniques demonstrates significant improvements across all evaluation metrics. Notably, both mAP@0.5 and mAP@0.5:0.95 showed significant improvement, indicating that super-resolution processing effectively enhances the spatial detail representation of farmland boundaries, enabling the segmentation model to more accurately identify the structure of farmland boundaries. At the same time, compared with the 1 m resolution reference image, the SR 1 m resolution image still exhibits a certain gap in recognition performance. This is primarily due to the lack of high-frequency information in the resampled 4 m resolution image and the inevitable detail deviations during the super-resolution reconstruction process. Nevertheless, overall, the segmentation results obtained from the SR 1 m resolution image have successfully restored the primary spatial structure of farmland boundaries, with recognition performance significantly superior to that of the resampled 4 m resolution image. This validates the effectiveness of super-resolution technology in enhancing the agricultural application value of medium-resolution remote sensing images.

### 3.3. Comparison Between Two Mainstream Super-Resolution Models

**Figure 8:**
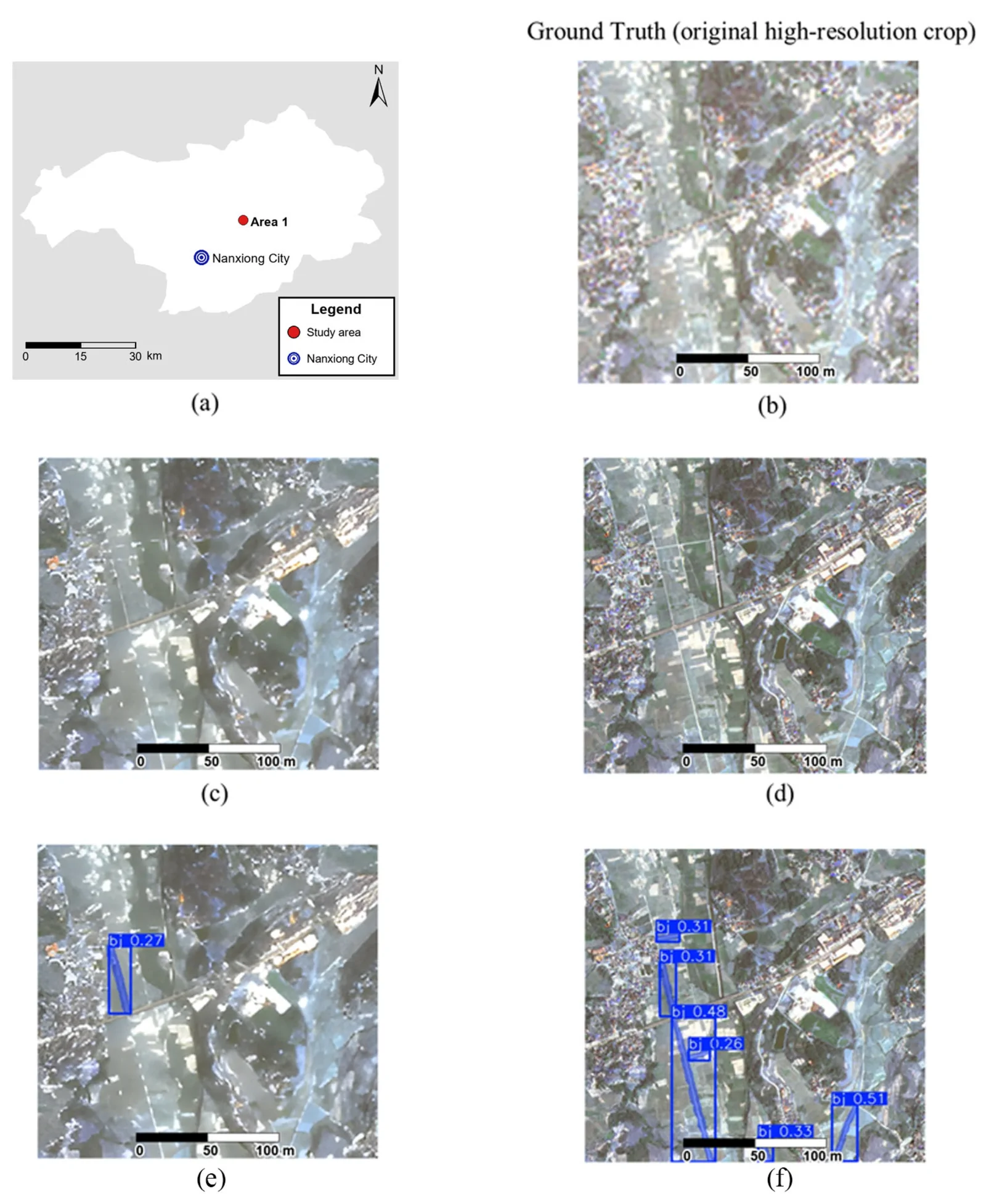
Comparison of Farmland Boundary Segmentation Performance Between Two Models. (**a**) EDSR + OpenCV reconstructed 1 m image of the study area; (**b**) Real-ESRGAN reconstructed 1 m image of the same area; (**c**) farmland boundary identification on the EDSR + OpenCV image; (**d**) farmland boundary identification on the Real-ESRGAN image; (**e**) detection bounding-box outputs on the EDSR + OpenCV result; (**f**) detection bounding-box outputs on the Real-ESRGAN result. Reported PSNR/SSIM values (EDSR + OpenCV: 24.19 dB/0.7543; Real-ESRGAN: 26.19 dB/0.7676) were computed on the 2400-tile SR-stage reconstruction held-out subset described in Section 3.1.1; re-reporting on a dedicated SR-stage test partition was not conducted in this revision because the SR model was not retrained for this purpose, and this design choice is documented as a limitation in Section 4.3.

This study compares the performance of the EDSR + OpenCV classical super-resolution model and the Real-ESRGAN super-resolution model in delineating farmland boundaries (Figure 8). Based on the numerical comparison of the two models, the EDSR + OpenCV classical super-resolution model achieved a mean PSNR of 24.19 dB and a mean SSIM of 0.7543, while the Real-ESRGAN super-resolution model achieved mean PSNR and SSIM values of 26.19 dB and 0.7676 (validation subset), respectively. It is evident that both the mean PSNR and SSIM values of the Real-ESRGAN super-resolution model are higher than those of the EDSR + OpenCV classic super-resolution model. In terms of the performance of the two models in extracting farmland boundaries, the EDSR + OpenCV classic super-resolution model identifies fewer farmland boundaries, while the Real-ESRGAN super-resolution model identifies more. The Real-ESRGAN super-resolution model outperforms the EDSR + OpenCV classic super-resolution model both numerically and visually.

## 4. Discussion

### 4.1. The Effectiveness of Super-Resolution Techniques in Enhancing Farm Boundaries

Combining Real-ESRGAN-based super-resolution reconstruction with farm boundary delineation provides a practical solution to address the inherent spatial limitations of medium-resolution satellite images. Experimental results demonstrate that the proposed workflow effectively bridges the gap between freely available GF-2 multispectral data (e.g., 4 m resolution) and the accuracy of 1 m resolution required for precise agricultural parcel mapping. Quantitative metrics (average *PSNR* of 26.19 dB and *SSIM* of 0.7676), computed on the validation subset, indicate that the method exhibits stable reconstruction performance in heterogeneous agricultural landscapes, consistent with previous findings that GAN-based super-resolution methods in remote sensing applications typically prioritize perceptual quality over pixel-level fidelity [75].

The high-order degradation modeling strategy adopted by Real-ESRGAN demonstrates significant advantages in agricultural remote sensing scenarios, as complex degradation processes such as atmospheric scattering, sensor noise, and compression artifacts severely degrade image quality [76]. Unlike traditional super-resolution methods that rely on simple bicubic downsampling assumptions [76], Real-ESRGAN’s blind recovery capability eliminates the need for paired low-to-high-resolution training data, thereby enhancing its adaptability to real satellite images with unknown degradation characteristics [77]. This feature aligns with the emerging paradigm of degradation-aware super-resolution networks, which explicitly model real-world blur kernels and noise distributions [48,78].

Visual evaluations demonstrate that super-resolution processing significantly improves boundary sharpness and structural continuity, particularly for linear features such as field boundaries, irrigation channels, and roads. These enhancements are crucial for downstream boundary segmentation tasks, as farmland boundaries typically manifest as subtle spectral-textural transitions rather than distinct edges [79,80]. The gradient-sensitive loss function modification implemented in this study’s training protocol effectively preserves high-frequency edge information, addressing the common issue where standard super-resolution models tend to oversmooth fine-scale details [81].

It should also be recognized that delineating terraced-field boundaries at a 1 m (or 0.8 m) ground sampling distance is an inherently extreme task: even at this resolution, a boundary of approximately 3 m occupies only a few pixels, and the effective boundary width approaches the sampling limit. Consequently, the achievable segmentation accuracy, although markedly improved by super-resolution, remains moderate rather than near-perfect; this intrinsic difficulty explains why the attained mAP values, while substantially higher than those of the 4 m baseline, are not exceptionally high.

### 4.2. Implications for Downstream Boundary Segmentation Tasks

The improvement in downstream performance observed in boundary segmentation based on YOLOv11x-seg confirms the hypothesis that super-resolution preprocessing serves as a functional enhancement rather than a mere visual improvement. The improved boundary continuity and reduced fragmentation in the segmentation output can be attributed to the enhanced spatial coherence provided by super-resolution images, which facilitates the reliable learning of more discriminative boundary features. The finding aligns with recent studies emphasizing the importance of task-driven super-resolution strategies, which specifically optimize reconstruction quality for subsequent analysis objectives [82,83].

A hybrid training approach incorporating multi-region agricultural samples is crucial for model generalization, particularly given the high morphological heterogeneity typical of smallholder agricultural systems. Experiments initially restricted to local training data exhibited significant boundary discontinuities and detection errors, reflecting the limited representativeness of single-region datasets for deep learning-based segmentation. Integrating diverse agricultural patterns—ranging from regularly arranged rice paddies to fragmented dryland plots—enabled the segmentation model to learn more robust boundary representations, thereby mitigating the domain transfer issues commonly encountered in agricultural remote sensing applications.

Notably, the performance improvement depends on the super-resolution preprocessing step, suggesting that enhanced spatial detail facilitates knowledge transfer across agricultural systems with significant geographical variations. The observation has important implications for operational mapping workflows in data-scarce regions, where access to a high-resolution reference image is limited. By enhancing the effective resolution of freely available medium-resolution data, the proposed method makes precision agriculture monitoring capabilities—previously limited to commercial high-resolution satellites—widely accessible.

### 4.3. Limitations of This Study

The super-resolution reconstruction process cannot recover missing spatial frequencies from the low-resolution image. In areas where land tenure patterns are highly fragmented or where boundaries are blurred due to vegetation cover, the reconstructed image exhibits local inconsistencies and minor artifacts, which propagate into segmentation errors [84]. These limitations reflect the inherent pathological characteristics of single-image super-resolution and underscore the method’s role as an enhancement strategy rather than a substitute for acquiring native high-resolution data.

The computational demands of super-resolution pose scalability challenges for continental-scale agricultural monitoring. Although the generator architecture of Real-ESRGAN employs efficient residual-dense blocks [75], processing large-scale satellite image archives requires substantial GPU resources and optimized inference workflows. Recent advancements in lightweight super-resolution architectures and model quantization techniques offer potential avenues for improving computational efficiency without significantly compromising performance [81].

Current evaluation frameworks primarily rely on geometric accuracy metrics (mAP, precision) and perceptual quality assessments (*PSNR*, *SSIM*). Future research should incorporate task-specific fidelity metrics that explicitly quantify the preservation of boundary topologies and the geometric accuracy of extracted parcel polygons, as these attributes are critical for cadastral applications and area estimation tasks. Furthermore, the integration of multi-temporal super-resolution techniques may leverage temporal redundancy in satellite image time series to further enhance reconstruction stability [48].

The generalization capability of the method proposed in this study across different sensor platforms (e.g., Sentinel-2, Landsat-9) and agro-ecological zones remains to be systematically validated. Recent studies on domain adaptation strategies for cross-sensor super-resolution and the development of sensor-agnostic degradation models represent promising directions for expanding the application of blind super-resolution techniques within the global agricultural monitoring framework [48].

Limitations. A more faithful simulation of medium-resolution GF-2 imagery would convolve the 0.8 m image with the sensor point-spread function (PSF) before sampling; such PSF/MTF-aware simulation is left for future work. The detection dataset, although geometrically non-overlapping at the tile level, is drawn from a single contiguous study area, so the train/val/test partitions are not geographically separated; spatial autocorrelation may inflate the reported metrics relative to a spatially separated test. Annotation was performed by a single expert annotator; inter-annotator agreement (Cohen’s κ) was not assessed because a second independent annotator is not part of this revision’s protocol, as documented in Section 2.3.2; the present single-annotator design is listed as a limitation here and is recommended for future work. The boundary-aware gradient loss (L_grad) introduced in Section 2.3.3 was not ablated in this revision: isolating its contribution would require re-training the super-resolution model under a matched compute budget, which exceeds the present budget; its contribution is therefore supported by the validation-set PSNR/SSIM behaviour reported in Section 3.1.1, and a controlled ablation is recommended as future work.

### 4.4. Future Work of This Study

Hardware/acquisition improvement: higher-resolution satellite platforms (e.g., sub-metre commercial sensors) and airborne imaging would alleviate the resolution bottleneck at the source. PSF/MTF-aware degradation simulation as noted above. Scene-level spatially disjoint evaluation: whole contiguous spatial blocks—not individual tiles—will be assigned to train/val/test partitions, and all metrics re-reported on that geographically separated held-out set.

## 5. Conclusions

This study proposed a super-resolution-assisted farmland boundary extraction framework for medium-resolution satellite images and integrated Real-ESRGAN image reconstruction with YOLO segmentation to enhance spatial details and improve farmland boundary detection accuracy.

Experimental results confirmed the effectiveness of the proposed framework: super-resolution reconstruction substantially improved farmland boundary segmentation accuracy over the resampled 4 m baseline, approaching the performance of the resampled 1 m reference, and outperformed the EDSR + OpenCV baseline both numerically and visually. These findings support the hypothesis that task-oriented super-resolution preprocessing serves as a practical, cost-effective enhancement for farmland boundary extraction from medium-resolution agricultural remote sensing imagery.

Future work will focus on integrating multi-temporal satellite images and advanced segmentation architectures to further improve farmland boundary extraction accuracy. Future research directions include: (a) integrating multi-source data fusion strategies that combine super-resolved optical images with synthetic aperture radar (SAR) observations to enhance all-weather monitoring capabilities; (b) developing task-specific super-resolution architectures explicitly designed for boundary preservation rather than general perceptual quality optimization; (c) implementing active learning frameworks to iteratively improve segmentation model performance with minimal human annotation requirements. Continued advancements in blind super-resolution technology, combined with increasingly sophisticated downstream analysis models, are expected to further bridge the gap between freely available Earth observation data and the accuracy requirements of modern precision agriculture.

## Figures and Tables

**Table 1:** Technical Parameters of Remote Sensing Image Data.

Data Attributes	Technical Parameters
Sensor	PMS1
Product Format	GeoTIFF
Product Serial Number	14523636001
Product Class	Level 1A
Product Type	Standard GF-2
Production Date	22 March 2025 01:03:12
Spatial Resolution	0.8 m (panchromatic, PAN)/3.2 m (multispectral, MS)
Coordinate System	WGS_1984
Scene Center	114.6° E, 25.3° N
Orbit Number	57567
Cloud Cover	0%
Launch Date	19 August 2014
Orbit Altitude	631 km
Revisit Period	5 days
Radiometric Resolution	10 bit

**Table 2:** Quantitative comparison of farmland boundary segmentation performance under different input image types.

Input Type	mAP@0.5	mAP@0.5:0.95	Improvement
resampled 4 m	0	0	—
SR 1 m	0.75502	0.62820	+0.755/+0.628
resampled 1 m	0.82311	0.73322	+0.823/+0.733

## Data Availability

Requests to access the datasets should be directed to the corresponding author. The super-resolution and detection pipeline was implemented in Python version 3.10 using the PyTorch version 2.7.1 deep-learning framework on an NVIDIA CUDA-enabled GPU (CUDA Version 12.8), building upon the official Real-ESRGAN repository, available at https://github.com/xinntao/Real-ESRGAN (accessed on 5 June 2025). The preprocessing, training, and inference scripts, together with the trained model configuration, are available from the corresponding author upon reasonable request.

## Supplementary Materials

The following supporting information can be downloaded at: https://www.mdpi.com/article/10.3390/s26175613/s1.
